# Environmental footprint family to address local to planetary sustainability and deliver on the SDGs

**DOI:** 10.1016/j.scitotenv.2019.133642

**Published:** 2019-11-25

**Authors:** Davy Vanham, Adrian Leip, Alessandro Galli, Thomas Kastner, Martin Bruckner, Aimable Uwizeye, Kimo van Dijk, Ertug Ercin, Carole Dalin, Miguel Brandão, Simone Bastianoni, Kai Fang, Allison Leach, Ashok Chapagain, Marijn Van der Velde, Serenella Sala, Rana Pant, Lucia Mancini, Fabio Monforti-Ferrario, Gema Carmona-Garcia, Alexandra Marques, Franz Weiss, Arjen Y. Hoekstra

**Affiliations:** a European Commission, Joint Research Centre (JRC), Ispra, Italy; b Global Footprint Network, 18 Avenue Louis-Casai, 1219 Geneva, Switzerland; c Senckenberg Biodiversity and Climate Research Centre (SBiK-F), Senckenberganlage 25, 60325 Frankfurt am Main, Germany; d Vienna University of Economics and Business (WU), Institute for Ecological Economics, Welthandelsplatz 1, 1020 Vienna, Austria; e Food and Agriculture Organization of the United Nations, Animal Production and Health Division, Viale delle Terme di Caracalla, 00153 Rome, Italy; f Animal Production Systems group, Wageningen University & Research, PO Box 338, 6700 AH Wageningen, the Netherlands; g Teagasc – Crops, Environment and Land Use Programme, Johnstown Castle, Wexford, Ireland; h European Sustainable Phosphorus Platform (ESSP), Avenue du Dirigeable 8, 1170 Brussels, Belgium; i Institute for Sustainable Resources, Bartlett School of Environment, Energy and Resources, University College London, WC1H 0NN London, UK; j KTH – Royal Institute of Technology, Department of Sustainable Development, Environmental Science and Engineering, Stockholm SE-100 44, Sweden; k Ecodynamics Group – Department of Earth, Environmental and Physical Sciences, University of Siena, Pian dei Mantellini 44, 53100 Siena, Italy; l School of Public Affairs, Zhejiang University, 310058 Hangzhou, China; m Department of Natural Resources, The Environment and The Sustainability Institute, University of New Hampshire, Durham, NH, USA; n University of Free State, 205 Nelson Mandela Dr, Park West, Bloemfontein 9301, South Africa; o Twente Water Centre, University of Twente, P.O. Box 217, Enschede, Netherlands; p Institute of Water Policy, Lee Kuan Yew School of Public Policy, National University of Singapore, Singapore; q R2Water Research and Consultancy, Amsterdam, Netherlands

**Keywords:** Footprint, Environmental footprint, Environmental footprint assessment, Family, Footprint family, Planetary boundaries

## Abstract

The number of publications on environmental footprint indicators has been growing rapidly, but with limited efforts to integrate different footprints into a coherent framework. Such integration is important for comprehensive understanding of environmental issues, policy formulation and assessment of trade-offs between different environmental concerns. Here, we systematize published footprint studies and define a family of footprints that can be used for the assessment of environmental sustainability. We identify overlaps between different footprints and analyse how they relate to the nine planetary boundaries and visualize the crucial information they provide for local and planetary sustainability. In addition, we assess how the footprint family delivers on measuring progress towards Sustainable Development Goals (SDGs), considering its ability to quantify environmental pressures along the supply chain and relating them to the water-energy-food-ecosystem (WEFE) nexus and ecosystem services. We argue that the footprint family is a flexible framework where particular members can be included or excluded according to the context or area of concern. Our paper is based upon a recent workshop bringing together global leading experts on existing environmental footprint indicators.

## Highlights

• We define a family of environmental footprints.
• We identify overlaps between different footprints.
• We analyse how they relate to the nine planetary boundaries.
• We discuss the relation with SDGs, WEFE nexus and ecosystem services.
• We argue that the footprint family is a flexible framework.

## Graphical abstract

**Figure:**
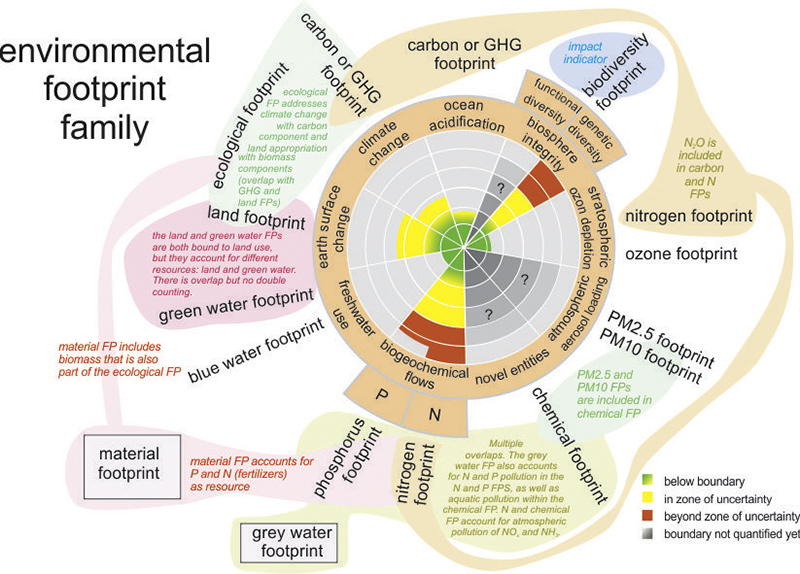

## 1 Introduction

**Fig. 1:**
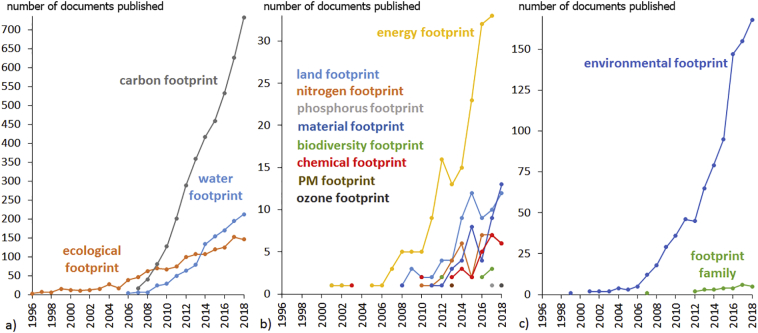
Number of documents published (Y-axis) on environmental footprints from 1996 to 2018 (X-axis) in Science Citation Index Expanded (SCI-EXPANDED) or Social Sciences Citation Index (SSCI). Footprints are depicted in different panels due to the different magnitude of the number of documents: (a) the three most published footprints; (b) other footprints with less publications and (c) umbrella terms “environmental footprint” and “footprint family”. Publications using terms close to “footprint”, such as “embedded resource” or “virtual resource”, are omitted.

Since the introduction of the first footprint metric, the ecological footprint in 1996 (Wackernagel and Rees, 1996), many other footprints have emerged in the literature (Galli, 2015a) and the number of papers with the topic “footprint” has been growing steadily (Fig. 1). Most of those papers have focussed on carbon (Wiedmann and Minx, 2008), water (Hoekstra and Mekonnen, 2012) and ecological (Wackernagel et al., 2002) footprints. Other footprints, with less publications until today, include the land (Kastner et al., 2012; O'Brien et al., 2015; Weinzettel et al., 2013), nitrogen (Galloway et al., 2014; Leach et al., 2012; Oita et al., 2016), phosphorus (Wang et al., 2011), material (Giljum et al., 2015, Giljum et al., 2016; Wiedmann et al., 2015), biodiversity (Lenzen et al., 2012), chemical (Hitchcock et al., 2012; Sala and Goralczyk, 2013) PM_2.5_ (Yang et al., 2018), PM_10_ (Moran et al., 2013), ozone (Meyer and Newman, 2018) and energy (Onat et al., 2015; Wiedmann, 2009) footprints.

The term “environmental footprint” is an umbrella term for the different footprint concepts that have been developed during the past two decades (Fang et al., 2016; Hoekstra and Wiedmann, 2014). The terminology is also used in the Life Cycle Assessment (LCA)-based product and organisation environmental footprint of the European Commission (EC, 2013).

Despite the growing interest around footprint indicators, relatively little research has focussed on integrating multiple footprints, which can together be referred to as the “footprint family” (Fang et al., 2014, Fang et al., 2016; Galli et al., 2012; Leach et al., 2016). Only 28 papers were published on this topic by the end of 2018, dwarfed by the 6735 studies published on primarily individual footprints (Fig. 1).

For integrated environmental assessments, scientific analyses, policy formulation, integrated policy decisions, and understanding trade-offs, different environmental footprints need to be studied simultaneously (Dalin and Rodríguez-Iturbe, 2016; Galli et al., 2012; Wiedmann and Lenzen, 2018). For example, replacing fossil by bio-energy might reduce a carbon footprint but will inevitably increase land and water footprints (Mekonnen et al., 2016). Footprint-family analyses are particularly suited to account for such trade-offs. Here, we aim to define the environmental footprint family. We limit our discussion to environmental footprints, thus excluding footprints related to the two other pillars of sustainability, as few footprints addressing social and economic issues exist and, in most cases, they have unclear definitions and limited applications (Galli, 2015a).

The aim of our paper is to systematize the existing environmental footprints, and in doing so, to bring clarity into the crowded field of footprint studies. We identify overlaps between different footprint indicators, analyse how they relate to planetary boundaries (Rockstrom et al., 2009; Steffen et al., 2015), and identify whether they can measure progress towards achieving the Sustainable Development Goals (SDGs) and address the water-energy-food-ecosystem (WEFE) nexus.

A limited amount of papers on the footprint family have been published. Hoekstra and Wiedmann (2014) and Čuček et al. (2015) reviewed current environmental footprints and reviewed global estimates of footprint scores relative to planetary boundaries, without the consideration of local sustainability that requires specific environmental footprints to remain within local boundaries. Čuček et al. (2012) and Fang et al. (2016) focused on the typology of environmental, social and economic footprints, but did not relate them to monitoring progress towards the SDGs or the WEFE nexus. Galli et al. (2012) and Fang et al. (2014) constituted different sets of a footprint family and called for a shift of focus from assessing single footprints in isolation to integrating diverse footprints from a systemic perspective, but both of them included only few footprints. The main added value of this paper is the systematization of the environmental footprint family and the discussion of its role in addressing local to planetary sustainability, measuring progress towards the SDGs and analyzing the WEFE nexus. Our paper is based upon a recently organized workshop at the Joint Research Centre in Ispra, Italy, which brought together, for the very first time, 23 global leading experts on existing footprint indicators, from 17 different institutions.

For clarity, Table 1 shows a list of the acronyms we use.

**Table 1:** Acronyms with definition.

Acronym	Definition
EC	European Commission
EE-MRIO	Environmentally-extended multi-regional input-output
EFA	Environmental footprint assessment
ES	Ecosystem services
FP	Footprint
gha	Global hectares
GHG	Greenhouse gases
HANPP	Human appropriation of net primary production
IEAG-SDGs	Inter-Agency Expert Group on SDG indicators
LCA	Life cycle assessment
LCI	Life cycle inventory
LCIA	Life cycle impact assessment
N	Nitrogen
OEF	Organisation environmental footprint
P	Phosphorus
PEF	Product environmental footprint
PM	Particulate matter
SDG	Sustainable Development Goal
UN	United Nations
WEF nexus	Water-energy-food nexus
WEFE nexus	Water-energy-food-ecosystem nexus

## 2 Systematization of footprints in the context of environmental concerns and local to planetary boundaries

### 2.1 Environmental footprints

Footprints are indicators of pressure of human activities on the environment. Footprint quantification is based on life cycle thinking along the whole supply chain (from producer to consumer, and sometimes to waste management) and aims to give a comprehensive picture of the quantified pressure. Each footprint focuses on a particular environmental concern, and measures either resource appropriation or pollution/waste generation, or both (Hoekstra and Wiedmann, 2014).

Footprints quantify pressure along the supply chain, with as basis unit footprints (Hoekstra and Wiedmann, 2014). A “unit footprint” is the footprint of a single process or activity and forms the basic building block for the footprint of a product, consumer, or producer or for the footprint within a certain geographical area. As such, footprints can be quantified for products at any stage of the supply chain, for companies or economic sectors. They can also be used for individuals or communities (as end consumers) or from the smallest geographical areas (such as streets or villages) up to the global level. This provides communication with a broad variety of stakeholders, from civil society individuals to industrial stakeholders and decision makers, up to policy makers (Hoekstra and Wiedmann, 2014).

**Fig. 2:**
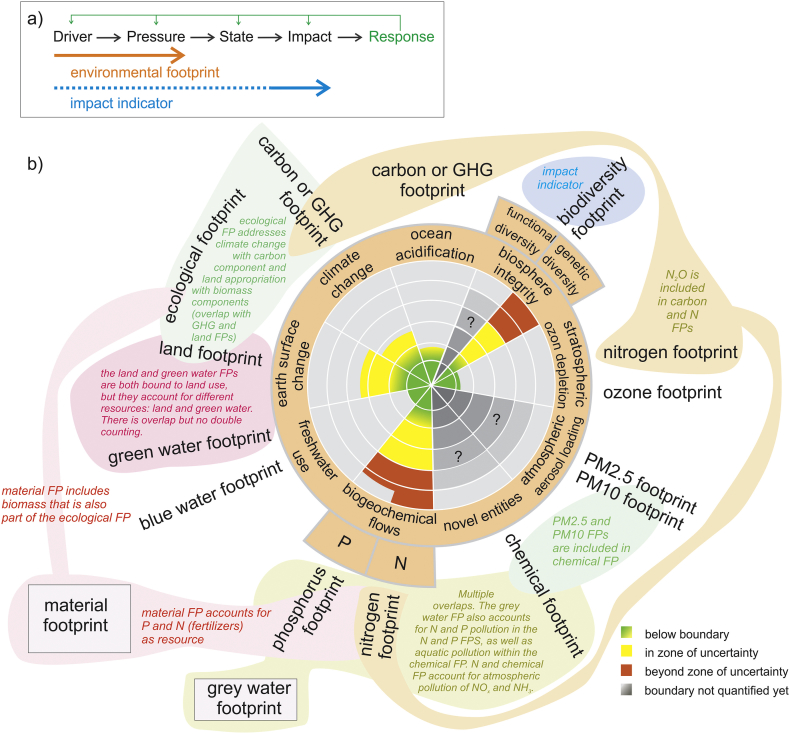
a) Linear representation of the DPSIR framework (drivers, pressure, state, impact and response) (OECD, 2003) and its theoretical relationship with environmental footprints and impact indicators. Since recently, some authors (Verones et al., 2017) also use the terminology “impact footprints” as relating to impact indicators, in addition to the pressure-related footprints we describe here. b) Correspondence of existing footprint indicators with the nine planetary boundaries, with visualization of overlap between different footprints. Fang et al. (2015) already included chemical pollution as planetary boundary (novel entity) with related chemical footprint. The material and grey water footprints do not correspond directly to a planetary boundary. FP=Footprint.

Environmental Footprint Assessment (EFA) and Life Cycle Assessment (LCA) are both based upon life cycle thinking but differ in aim and approach. Environmental footprints are resource use and emissions oriented, combined referred to as *pressure* oriented, whereas LCA is *impact* oriented. Pressure indicators are different from impact indicators, as they inform users on the pressure human activities place on ecosystems (e.g., the land used to produce a crop) rather than on the potential consequences (impact) due to such pressure (Fig. 2a). Some footprints, such as the water footprint, however, can include an impact phase in their full assessment (Hoekstra et al., 2011). Here, we focus on environmental footprints as employed in EFA, not their uptake and use in LCA.

### 2.2 Planetary boundaries

Rockstrom et al. (2009) and Steffen et al. (2015) identified nine critical processes that regulate the Earth system functioning. For each of these critical processes, they proposed a main control variable and defined boundaries that should not be exceeded to keep the Earth system in a safe operating space, recognizing though the complexity of the Earth System and the interaction between critical processes. In a preliminary assessment, Steffen et al. (2015) found that, due to human activities, four of these boundaries are violated: climate change, biosphere integrity, biogeochemical flows (nitrogen and phosphorus), and land system change, whereby the perturbations of biogeochemical flows and genetic diversity are even beyond the zone of uncertainty. Research on planetary boundaries is in its infancy, so considerable progress is to be expected in this field in the near future.

Environmental footprint indicators measure natural resources use and emissions while the planetary boundaries provide levels of perturbation that are believed to ensure that the Earth System is kept in Holocene-like conditions that are favourable for humanity. It is possible to reconcile the two and show how the existing footprint indicators could be used to measure the extent to which Earth System processes are being disturbed by human activities and thus planetary boundaries approached.

### 2.3 Systematization and relationship with planetary boundaries

Environmental footprints measure either resource use or emissions, or both (Table 2). In the first case, they account for the amount of resources used to produce the goods and services human societies consume; in the second case, they account for the amount of pollutants emitted to the environment due to human production and consumption activities (Fang et al., 2016).

**Table 2:** Framework for the systematization of footprints, based on their environmental concern and scope (measuring resource use/emissions) (first four columns), identification of overlaps (column 5) and descriptive relationships between existing environmental footprints and the nine planetary boundaries (columns 6 and 7). A distinction is made between planetary boundaries and local thresholds. The footprints can show which human activities contribute to what degree to reaching or transgressing the global planetary boundary or local thresholds. FP=Footprint.

Environmental concern	Pressures		Impacts	Overlaps	Planetary boundary	Local thresholds
	Resource use	Emissions				
Climate change and ocean acidification	Carbon component of the ecological FP	Carbon FP (anthropogenic greenhouse gas emissions)		The N_2_O emissions component is included in both the carbon and nitrogen FP. Land for CO_2_ sequestration is included in ecological FP	In Steffen et al. (2015), the global boundary is set at 350 ppm CO_2_ in the atmosphere, which relates to a maximum acceptable level of global warming, and can be translated back to a maximum acceptable carbon FP.	Not applicable
					Maximum level of ocean acidification (resulting from CO_2_), to be translated back to a maximum acceptable carbon footprint	Not applicable
Water scarcity and water pollution	Green and blue water FP	Grey water FP	Blue water stress and water pollution, the second stage in water FP assessment	The nitrogen and phosphorus related grey water FPs are also represented in the nitrogen and phosphorus FPs, respectively. The chemical FP accounts for aquatic pollution	Blue water FP: Limited aggregate global accessible blue water availability Green water FP: Limited aggregate global green water availability, as proposed by Schyns et al. (2019)	Limited monthly blue and green water availability per catchment; limited assimilation capacity for grey water FP
Land appropriation/availability	Land FP biomass components of the ecological FP			Land FP is part of ecological FP Green water FP is bound to land use, but accounts for different resource	In Steffen et al. (2015), the global threshold is defined at 75% of original forest cover remaining for three biomes (tropical, temperate, boreal), calculated as a weighted average of the boundaries per biome.	Limited bioproductive area per biome or ecoregion
Nitrogen use and pollution	Nitrogen input FP, used by some authors (Vanham et al., 2015)	Nitrogen FP (total losses of N to the environment, including reactive nitrogen compounds (NH_3_, NO_x_, N_2_O, nitrates, and organic nitrogen) and N_2_).		Nitrogen water pollution is represented in the grey water FP. The component N_2_O is included in both the carbon and nitrogen FPs. Nitrogen and chemical FPs account for aquatic N pollution as well as atmospheric pollution of NO_x_ and NH_3_ Nitrogen and ozone FP are complementary, as they account for different ozone depleting gases	Limited aggregated assimilation capacity	Limited assimilation capacity of the environment for reactive N losses to water bodies per catchment and to the atmosphere
					Maximum level of acceptable stratospheric ozone depletion, to be translated back to maximum N_2_O emission	Not applicable
Phosphorus use and pollution	Phosphorus input FP	Phosphorus to water bodies FP		Phosphorus water pollution is represented in the grey water FP. Phosphorus and chemical FPs account for aquatic P pollution	Limited aggregated assimilation capacity	Limited assimilation capacity of the environment for P pollution per catchment
Biodiversity loss			Indicator “biodiversity loss”, often referred to as biodiversity footprint	Biodiversity loss is a result of different pressures (FPs)	Global biosphere integrity (genetic, functional diversity)	Local biosphere integrity (genetic, functional diversity)
Chemical pollution		Chemical FP (emission of chemical substances into water, air or soil)	Certain approaches quantify impact (Zijp et al., 2014)	Water related pollution is also represented in the grey water FP. Nitrogen and chemical FPs account for aquatic N pollution as well as atmospheric pollution of NO_x_ and NH_3_ Chemical FP includes PM_2.5_ and PM_10_ pollution	Limited aggregated assimilation capacity Would fit under “novel entities”	Limited assimilation capacity of the environment for chemical pollution per catchment, to the soil and the atmosphere Would fit under “novel entities”
Particulate concentration of aerosols in the atmosphere		PM_2.5_ and PM_10_ FPs		PM_2.5_ and PM_10_ pollution are included in chemical FP	Atmospheric aerosol loading	Atmospheric aerosol loading
Ozone depletion		Ozone FP (Meyer and Newman, 2018)		Ozone and nitrogen FP are complementary, as they account for different ozone depleting gases	Maximum level of acceptable stratospheric ozone depletion, to be translated back to maximum ozone-depleting gas emissions	Not applicable
Material extraction	Material FP (EUROSTAT, 2018) (use of materials: fossil fuels, metal ores, minerals, biotic resources)			Material FP accounts for P and N fertilizer use (resource use component of P and N FPs) Material FP includes biomass, also part of ecological FP Material FP includes fossil fuels as resource use, not as pollution. So no overlap with carbon FP.	Currently no planetary boundary identified, but proposed by some scholars for biomass	

Fang et al. (2015) presented a preliminary thematic matching of some environmental footprints and planetary boundaries, and concluded multiple matchings. This is due to overlaps between different footprints, a matter we analyse in detail here as listed in Table 2 and visually presented in Fig. 2b.

Earth system processes operate across scales, from local catchments or biomes up to the level of the earth system as a whole. The focus of environmental footprints on resources use and emissions caused by human activities makes them relevant also for assessing local processes. While the estimation of planetary boundaries by Rockstrom et al. (2009) was based on global analyses, the authors recognized that the control variables for many processes are spatially heterogeneous. Steffen et al. (2015) therefore refined the methodology and developed global planetary boundaries taking into account also regional-level boundaries. Planetary boundaries, which are based on regional assessment, are biodiversity integrity, freshwater use, earth surface change (land use change), biogeochemical flows and atmospheric aerosol loading (Fig. 2b and Table 2). The planetary boundaries for stratospheric ozone depletion, ocean acidification and climate change are only relevant at a global scale, although the related impacts can be locally very different.

The carbon footprint (or greenhouse gas footprint (Čuček et al., 2015)) is an emission footprint, which measures the emission of greenhouse gases (GHG) such as carbon dioxide (CO_2_), methane (CH_4_) and nitrous oxide (N_2_O) to the atmosphere. Conceptually the carbon footprint also includes GHG emissions from land-use change, although in practice this is not always the case.

The water footprint measures both the consumption of fresh water as a resource and the use of fresh water to assimilate waste, where the latter component is referred to as grey water footprint (Hoekstra and Mekonnen, 2012). Water resources include both blue and green water (Rockström et al., 2009).

The ecological footprint measures the appropriation of land to both produce renewable biomass resource and uptake waste via CO_2_ sequestration (Borucke et al., 2013). These demands are expressed in bioproductive land-equivalent units (expressed in global hectares or gha) (Galli, 2015b) and compared with the bioproductive hectare-equivalents available within a given territory to provide insights on a given country's over or under use of its ecological assets' regenerative capacity (Wackernagel et al., 2002).

The land footprint measures the amount of land required for the supply of food, materials, energy and infrastructure, expressed in physical hectares (MacDonald et al., 2015; Thomas et al., 2014) (or km^2^) or equivalent land units (i.e. global hectares) (Wackernagel et al., 2002; Weinzettel et al., 2013).

Nitrogen and phosphorus are essential nutrients for all living organisms, but their abundant utilization for human prosperity contributes to several environmental impacts such as climate change, eutrophication, acidification and biodiversity loss (Erisman et al., 2008; Leip et al., 2015; Sutton et al., 2011). The nitrogen footprint measures the emissions of reactive N to the atmosphere and to water bodies. In several studies, the nitrogen footprint also includes emissions of N_2_, which does not contribute to any environmental pressure and does not depend on a scarce resource (Peñuelas et al., 2013), but gives a measure for the anthropogenic mobilization of nitrogen (Pelletier and Leip, 2014). The phosphorus footprint measures both the use of P as a resource and P losses to water bodies. The former is very relevant as exploitable P stocks (rock phosphate) are limited (Obersteiner et al., 2013; van Dijk et al., 2016). The release of P from soils to the hydrosphere depends on several factors, in particular the soil type, which might be able to bind a large share of P input and make it unavailable for both plant uptake and environmental losses (Zhang et al., 2017).

The chemical footprint (Hitchcock et al., 2012; Sala and Goralczyk, 2013) accounts for all chemical substances released into the environment which may ultimately lead to ecotoxicity and human toxicity impacts. A list of chemical substances is exhaustive, including pesticides or heavy metals.

The PM_2.5_ (Yang et al., 2018) and PM_10_ (Moran et al., 2013) footprints measure particulate matter pollution to the atmosphere. These are also included in the chemical footprint.

The ozone footprint (Meyer and Newman, 2018) proposed by Meyer and Newman measures emission of gases controlled or due to be controlled under the Montreal Protocol in terms of ozone depleting potential weighted kilograms. As N_2_O, a major ozone-depleting gas, is not included in this protocol, this component of the nitrogen footprint is complementary to the ozone footprint in addressing the planetary boundary stratospheric ozone depletion.

The material footprint (Wiedmann et al., 2015) measures the use of materials from a consumption perspective, allocating all globally extracted and used raw materials to domestic final demand (Giljum et al., 2015). It encompasses four material categories: metal ores, non-metallic minerals, fossil fuels and biomass (crops, crop residues, wood, wild fish catch, etc.). Material Footprint and other Material Flow-based indicators have been widely used to support and monitor resource efficiency policy internationally. This is the case, for instance, of the EU Resource Efficiency Initiative (Demurtas et al., 2015; EC, 2011).

Biodiversity loss measures impact as a result of different pressures, such as land and water use or chemical pollution. Work on the biodiversity footprint is relatively young (e.g. Kitzes et al. (2017), Lenzen et al. (2012)) and no common unit of measure exists. Given the multiple dimensions and complexities of biodiversity, a range of units will be needed for a comprehensive picture of how consumption drives biodiversity loss (Marques et al., 2017).

Only in few cases, the currently proposed control variables of Steffen et al. (2015) are identical to environmental footprints. Regarding the planetary boundary freshwater use, the global control variable “maximum amount of consumptive blue water use” is identical to the blue water footprint. The basin control variable, “blue water withdrawal as percentage of mean monthly river flow”, is identical to the water footprint, apart from the fact that the water footprint quantifies consumptive water use and not water withdrawal. An unresolved issue in footprint studies so far is that of groundwater abstraction and use, and the associated groundwater depletion, although recent work has quantified groundwater depletion associated with agricultural products globally (Dalin et al., 2017).

For some footprints, thresholds for local environmental problems seem to be an equally relevant application as are planetary boundaries. For freshwater use, for example, Mekonnen and Hoekstra (2016) quantified local maximum blue water footprints based upon blue water stress for grid cells of 30 × 30 arc min.

While the planetary boundaries framework does not explicitly include materials, the definition of a safe operating space for material resource use has been widely discussed in the literature. For instance, targets for biotic and abiotic resource consumption are proposed in Bringezu (2015), Dittrich et al. (2012) and Mudgal et al. (2012) using the concept of human appropriation of net primary production (HANPP). Haberl et al. (2014) discuss upper limits of yearly biomass flows, which could support the planetary boundaries assessment.

In the interpretation of results related to the various planetary boundaries (for example like in Fig. 2b), it is important to keep in mind that the planetary boundaries have not been designed to be used directly in a comparative context. Caution is appropriate when assessing the relevance and urgency to tackle boundary issues based on simply quantitatively comparing the results. For example, a 20% overshoot for one boundary does not necessarily mean it has to be less relevant than a 40% overshoot related to another boundary. Steffen et al. (2015) argue that two planetary boundaries – namely climate change and biosphere integrity – have each the potential to push the Earth system out of the safe operating space alone. However, due to the complex Earth system dynamics with feedbacks and interactions across all critical processes, only the safeguarding of all planetary boundaries can ensure that the Earth system remains in the Holocene state.

### 2.4 Footprint terminology in other indicators

Other indicators use the terminology footprint and are by their authors generally regarded as such, including the energy (Onat et al., 2015; Wiedmann, 2009) and emergy (Bastianoni et al., 2008; Odum, 1988) footprints. The energy footprint is both expressed as the carbon component of the ecological footprint (Mancini et al., 2016; Wiedmann, 2009) or the amount of energy consumed along the supply chain (Onat et al., 2015). The emergy footprint relates to the latter and deals with embedded primary solar energy equivalents, also referred to as “solar energy footprint”. Other related terminologies include the cumulative energy demand and embodied energy. The energy footprint in its variant of measuring use of energy (Onat et al., 2015) as well as the emergy footprint, do not correspond to a planetary boundary, because energy availability in itself has not been considered thus far as a planetary boundary given the large amount of solar energy that the earth is receiving, which can potentially be converted.

The terminology is also used in the Life Cycle Assessment (LCA)-based product and organisation environmental footprint of the European Commission (EC, 2013). More particularly, the terminologies Product Environmental Footprint (PEF) and Organisation Environmental Footprint (OEF) are used. Their overarching purpose is seeking to reduce the environmental impacts of goods and services (PEF) and organisations (OEF), respectively, taking into account the whole supply chain, as multi-criteria measures. As LCA measures, they include a life cycle inventory (LCI) and life cycle impact assessment (LCIA) phase. As such, they can be regarded as complementary indicators to the footprint family we describe here. In the LCIA phase, the PEF and OEF use more than 15 different impact categories, including (aquatic fresh water) ecotoxicity and human toxicity (cancer and non-cancer effects) (EC, 2013; Sala et al., 2019). Each impact category is using specific indicators of impact. For example for ecotoxicity, the indicator could be expressed in cumulative toxic units, namely the result of the multiplication of the mass -resulting from a fate modelling of the chemical emitted in a certain compartment- by the exposure potential and the toxicity exerted by the chemical. This allows highlighting which chemicals have the potential to contribute the most to the overall impact.

As environmental footprints quantify pressure (resource use and/or pollution), they do not quantify human and ecotoxicity. In a further impact assessment phase, environmental footprints can contribute to address human and ecotoxicity.

## 3 Environmental footprints and Sustainable Development Goals (SDGs)

In September 2015, heads of United Nations member states from around the world adopted the 2030 Agenda for Sustainable Development, consisting of 17 SDGs and 169 targets, monitored by means of 230 individual indicators. These indicators, identified and proposed by the Inter-Agency Expert Group on SDG indicators (IEAG-SDGs), were agreed upon by the 47th Session of the UN Statistical Commission in March 2016. Of the different environmental footprints, the material footprint is the only one included as an official SDG indicator (number 8.4.1 as well as 12.2.1 and 12.2.2), although a few other SDG indicators relate directly to other environmental footprint indicators (Table 3). Indicator 11.6.2 for example accounts for annual mean levels of fine particulate matter (e.g. PM2.5 and PM10) in cities (population weighted) and thereby directly relates to the PM2.5 and PM10 footprints. However, these footprints measure particulate matter pollution to the atmosphere (Table 2), and are therefore not identical to indicator 11.6.2. Many SDG indicators relate indirectly to the environmental footprint indicators, but these are not discussed as the list would be too elaborate. As an example, all footprint indicators deal/relate with SDG 12 on sustainable consumption and production due to their producers and consumer approach, but among SDG12 indicators, apart from 12.2.1 and 12.2.2, none relate directly to particular footprints. In addition, all footprint indicators relate to target 8.4 on the improvement to global resource efficiency in consumption and production.

**Table 3:** Representation of environmental footprints in SDGs, SDG targets and SDG indicators.

Footprint	SDG	SDG target	Official SDG indicator	Relates to SDG indicator	Comments
Carbon footprint	SDG 9 “industry, innovation and infrastructure”	9.4		9.4.1 CO2 emission per unit of value added	The carbon footprint can be measured from a value-added perspective (Fang and Heijungs, 2014)
	SDG 13 “climate action”				The indicators of this SDG do not relate to GHG emissions (thus carbon footprint) directly
Water footprint	SDG 6 “clean water and sanitation”	6.3 6.4		6.4.1 Water productivity 6.4.2 Level of water stress	The grey WF measures progress regarding target 6.3 (Hoekstra et al., 2017); The blue WF measures progress towards target 6.4. In a WF assessment, blue water stress is quantified along the supply chain. In order to be in line with the SDGs, indicator 6.4.2 should be used. (Vanham et al., 2018c). A WF quantifies net water withdrawal, not gross
Ecological footprint, land footprint	SDG 15 “life on land”	15.1		15.1.1 Forest area as a proportion of total land area	
		15.3		15.3.1 Proportion of land that is degraded over total land area	
	SDG 11 “sustainable cities and communities”	11.3		11.3.1 Ratio of land consumption rate to population growth rate	The target aims to limit land expansion of growing cities, recognizing that land is needed for agriculture and ecosystem services
Nitrogen footprint, phosphorus footprint	SDG 6 “clean water and sanitation”	6.3		6.3.1 Proportion of wastewater safely treated	
				6.3.2 Proportion of bodies of water with good ambient water quality	
	SDG 14 “life below water”	14.1		14.4.1 Index of costal eutrophication and floating plastic debris density	Target 14.1: by 2025, prevent and significantly reduce marine pollution of all kinds, in particular from land-based activities, including marine debris and nutrient pollution
Material footprint	SDG 8 “decent work and economic growth”	8.4	8.4.1 Material footprint, material footprint per capita, and material footprint per GDP		Indicator to reach target 8.4: Improve progressively, through 2030, global resource efficiency in consumption and production and endeavour to decouple economic growth from environmental degradation. All footprint indicators relate to target 8.4 on the improvement to global resource efficiency in consumption and production.
	SDG 12 “responsible production and consumption”	12.2	12.2.1 Material footprint, material footprint per capita, and material footprint per GDP		Material footprint is also a key indicator in achieving responsible production and consumption. All footprint indicators deal/relate with SDG 12 on sustainable consumption and production due to their producers and consumer approach.
			12.2.2 Domestic material consumption, domestic material consumption per capita, and domestic material consumption per GDP		
Biodiversity footprint	SDG 14 “life below water”	14.4		14.4.1: Proportion of fish stocks within biologically sustainable levels	
		14.5		14.5.1 - Coverage of protected areas in relation to marine areas	
	SDG 15 “life on land”	15.1		15.1.2: Proportion of important sites for terrestrial and freshwater biodiversity that are covered by protected areas, by ecosystem type	
		15.4		15.4.1: Coverage by protected areas of important sites for mountain biodiversity	
		15.5		15.5.1 Red list index	
PM_2.5_ and PM_10_ footprint	SDG 11 “sustainable cities and communities”	11.6		11.6.2 Annual mean levels of fine particulate matter (PM_2.5_ and PM_10_) in towns and cities (population weighted)	
Ozone footprint					Ozone is not accounted for in the SDG framework
Energy footprint; emergy footprint	SDG 7 “affordable and clean energy”	7.3		7.3.1 Energy intensity measured in terms of primary energy and GDP	

## 4 Environmental footprints and the water-energy-food-ecosystem (WEFE) nexus

**Fig. 3:**
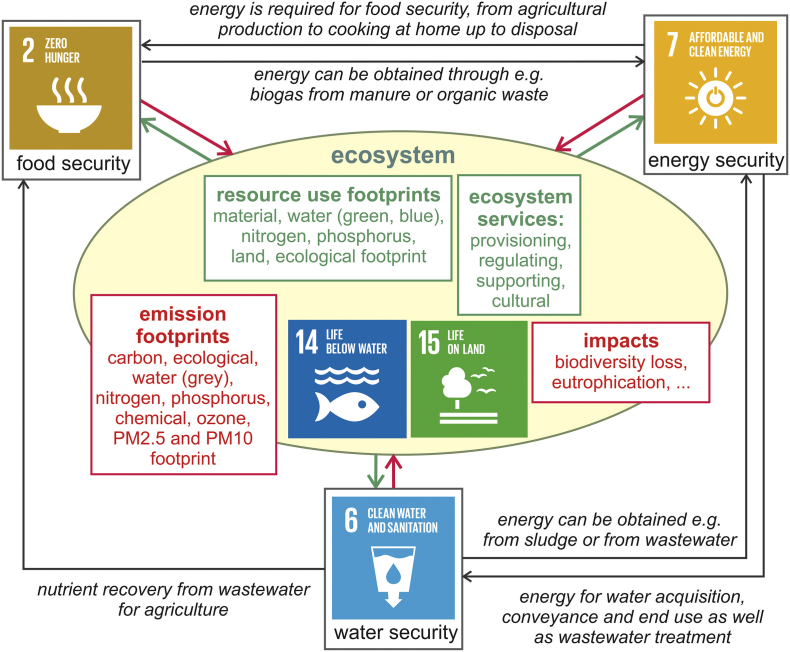
Graphical representation of the Water-Energy-Food-Ecosystem (WEFE) nexus, with representation of different environmental footprints of the footprint family. The green arrows represent resources and ecosystem services (ES) (where certain provisioning ES also relate to resources) required to provide the securities. The red arrows represent pollution and impacts on the ecosystem due to the provision of the securities. (For interpretation of the references to colour in this figure legend, the reader is referred to the web version of this article.)

The WEFE nexus (Fig. 3) is being recognized as a conceptual framework for achieving sustainable development (Biggs et al., 2015), including by international institutions like the UN (FAO, 2019) and the European Commission. It has become central to discussions regarding the development and subsequent monitoring of the SDGs. The WEFE nexus is a cross-sectoral perspective, which requires that response options go beyond traditional sectoral approaches. It means that the three sectors or securities — water security, energy security and food security (SDGs 6, 7 and 2) — are inextricably linked and that actions in one area more often than not have impacts in one or both of the others (Hoff, 2011; Vanham, 2016). Ecosystems are central in providing these three securities through the services (and resources) they provide. On the other hand, they are heavily affected by the process of providing these three basic human securities. Indeed, to achieve the SDGs, the important trade-offs and synergies of the WEFE nexus need to be accounted for.

Environmental footprints are indicators or tools that provide essential information for an analysis of the WEFE nexus (Fig. 3). A particular strength in their use is that they quantify pressure along the whole supply chain, up to the consumer level (potentially including the end of life level). The three securities relate to this consumer level, within a particular geographical setting (e.g. city, country) (Vanham, 2018). As it is recognized that local to global solutions for sustainable development need to come from measures at all stages along the supply chain (Foley et al., 2011; Godfray et al., 2010), the use of environmental footprints seems necessary. Indeed, many past footprint studies have considered the footprint of the full supply chain up to the consumer level. For example, consumer-level studies have assessed the footprints of healthy diets at different spatial scales: global (Chaudhary et al., 2018; Jalava et al., 2014, Jalava et al., 2016; Kastner et al., 2012), regional (Vanham et al., 2013), national (Galli et al., 2017; Vanham, 2013), city (Vanham et al., 2019) and even villages and city boroughs (Vanham et al., 2018). In addition, the reduction of consumer food waste and its impact on different footprints has been studied (Kashyap and Agarwal, 2019; Kummu et al., 2012; Vanham et al., 2015).

The concept of ecosystem services (ES) is complementary to the environmental footprint family. ES are necessary to provide the three securities, and by providing them, are in turn negatively affected. ES can be categorized in provisioning, regulating, supporting (maintenance) and cultural ES (EEA, 2019). Only certain provisioning ES relate directly or overlap with particular footprints (Table S1). These are the biomass components of the material and ecological footprints for the biotic provisioning ES of biomass, the blue water footprint for the abiotic provisioning ES of water and the material footprint for the abiotic provisioning ES of mineral resources. Other ES do not directly overlap with environmental footprints, although many are essential for the WEFE nexus such as the maintenance ES of pollination, which is important for food security but at risk due to decreases in insect populations (Sánchez-Bayo and Wyckhuys, 2019), among others as a result of the substantial chemical footprint of the food system (Jørgensen et al., 2018).

## 5 Application of the environmental footprint family

Recently, different footprint family assessments have been conducted. Springmann et al. (2018) e.g. analysed how the global food system can stay within environmental limits by evaluating five environmental footprints (carbon, land, blue water, nitrogen and phosphorus footprints) towards their planetary boundaries.

We present a comprehensive overview of the footprint family and identify overlaps. But we acknowledge both conceptual and methodological issues that require further research.

From a conceptual viewpoint, we acknowledge the existence of a currently unresolved dichotomy between the linearity of the DPSIR approach that underlies footprint thinking and the non-linear dynamics of complex systems, which are characterized by thresholds and abrupt change, slow and fast variables, surprises and strong nonlinearities, feedback loops, and bifurcations. Although it is quite difficult to relate a change in pressure on a system (e.g., the earth system) to the response by, or functioning of, the system, further research is needed to relate growing environmental pressures to complex dynamics. This means connecting drivers/pressures with responses and analyzing feedback loops (green arrows in Fig. 2a), rather than isolating them and leaning to a linear cause-effect thinking as currently done for ease in calculation. Collaboration is thus encouraged between earth system scientists and footprint accountants to shed light on the interconnections existing among the planetary boundaries, among footprint indicators and between them, and to understand how a system might respond, often in non-linear ways, to the pressures measured by footprint indicators.

From a methodological viewpoint, two key issues need to be highlighted and researched in the future. First, the planetary boundaries define nine critical earth system processes whose effective management is key to the maintenance of a resilient and accommodating state of the planet (i.e., humanity's safe operating space). They define the smallest set of critical, interacting processes that define the state of the earth system as whole; these control variables thus act as indicators for the functioning of a particular process, they assess the position or state of the control variable, and are global. Planetary boundaries can be translated to individual quota and combined with minimum resource requirements to fulfil basic needs; the space left between the maximum and minimum is called the safe and just operating space (Raworth, 2017). O'Neill et al. (2018) downscale four planetary boundaries (climate change, land-system change, freshwater use and biogeochemical flows) to per capita equivalents, and compare these to national consumption footprints (phosphorus, nitrogen, blue water, ecological and material footprints and eHANPP). They show how one can assess a country's performance relative to this “safe and just space”. Meyer and Newman (2018) propose to translate planetary boundaries to product level by showing how the consumption of a product contributes to a person's daily quota per planetary boundary.

Secondly, it must be acknowledged that footprint indicators have so far been calculated using different methodological approaches (Galli et al., 2013), yielding different results, which has been the subject of several analyses (Bruckner et al., 2015; Hubacek and Feng, 2016; Kastner et al., 2014; Tukker et al., 2016). These methods range from process-based or LCA approaches based on physical quantities and environmentally-extended multi-regional input-output (EE-MRIO) approaches based on economic proxies to hybrid approaches aimed to combine the advantages of both (Ewing et al., 2012). Further research is needed to streamline the calculation of the multiple footprints and bring them under a single accounting framework to enable results comparisons and trade-off assessment (Ewing et al., 2012; Galli et al., 2013). Ideally, multiple streamlined methods should be tested and their results further compared to identify the most reliable and informative methodology for footprint family assessments.

## 6 Conclusions

During the last two decades, many environmental footprints have been introduced, with an increasing amount of primarily single footprint assessments in the literature. The integration of these footprints into an environmental footprint family has received little focus in research. In this paper, we systematize existing footprints and propose a footprint family that provides a tool for environmental sustainability assessment, recognizing that this is a flexible framework, where particular members can be included or excluded according to the context or area of concern, and the trade-offs that are of relevance. Complex systems like the food system generally require the inclusion of many footprints, as the inclusion of a footprint like the chemical footprint, which accounts for pesticides, can give substantially different results when evaluating industrial and organic farmed systems.

Footprints quantify either resource use or emissions, or both. Many footprints show overlaps, and when conducting a footprint family assessment these overlapping components should be accounted for. Ideally these should also be presented as separate components. Apart from the material and grey water footprint, the carbon, blue and green water, ecological, land, nitrogen, phosphorus, PM_2.5_ and PM_10_, ozone, and biodiversity footprints provide information on eight of nine planetary boundaries. Chemical pollution is by different authors proposed as a “novel entity” planetary boundary, for which the chemical footprint can be a relevant indicator.

Environmental footprint indicators can be used to identify to what extent different processes and societies contribute to reaching or exceeding planetary boundaries, from local to global levels. We argue that environmental footprint indicators have largely added value to measuring the degree to which different processes contribute to reaching or exceeding planetary boundaries. An added value of the footprint approach is addressing not only to what extent we have reached certain boundaries, but also how different individual human activities and communities contribute to the overall footprints, as they account for the whole supply chain up to the consumer level, thereby identifying potential measures (diet shift, food waste reduction, changing the composition of the energy mix) how to reduce them. Since footprints are typically estimated as the sum of the footprints of different human activities and regions, they provide a basis for priority setting when footprints have to be reduced given that boundaries are exceeded.

Of all environmental footprints, only the material footprint is an official SDG indicator. The other footprints have direct or indirect links to different other SDG indicators, spread over different SDG targets. Ozone and thereby the ozone footprint is not represented in the SDG framework. To achieve SDG 2 (food security), SDG 6 (water security) and SDG 7 (energy security) in an environmentally sustainable way, the WEFE nexus framework is essential to assess trade-offs and synergies between these closely interlinked sectors. Ecosystem services are also essential to provide the WEF securities, and are in turn negatively affected. Certain provisioning ES relate directly or overlap with the material, ecological and blue water footprints. Other ES do not directly overlap with environmental footprints.

Demand for water, energy and food is increasing, driven by a rising global population, rapid urbanization, changing diets and economic growth. We argue that the footprint family is a valuable tool to analyse the nexus, considering pressures along the entire supply chain. Indeed, as adaptation measures on the consumer side of the supply chain are also necessary to achieve the three primal human securities, footprints provide an important added value in their ability to quantify and communicate such consumer changes.

The following is the supplementary data related to this article.

Table S1 Direct relationship environmental footprints with relevant classes of provisioning ecosystem services, according to CICES (EEA, 2019). Table S1

## Conflict of interest

The authors declare no conflict of interest.
