# Supplementary material for: Environmental footprint family to address local to planetary sustainability and deliver on the SDGs
Source: Sci Total Environ. 2019 Nov 25;693:133642. doi: 10.1016/j.scitotenv.2019.133642 (PMC6853168; doi:10.1016/j.scitotenv.2019.133642)
Supplement: Table S1 — Direct relationship environmental footprints with relevant classes of provisioning ecosystem services, according to CICES (EEA, 2019). [file mmc1.docx]

Table S1 Direct relationship environmental footprints with relevant classes of provisioning ecosystem services, according to CICES(EEA, 2019)

| Section | Division | Group | Class | Footprint |
| --- | --- | --- | --- | --- |
| Provisioning (Biotic) | Biomass | Cultivated terrestrial plants for nutrition, materials or energy | Cultivated terrestrial plants (including fungi, algae) grown for nutritional purposes | Biomass component of material footprint and ecological footprint |
|  |  |  | Fibres and other materials from cultivated plants, fungi, algae and bacteria for direct use or processing (excluding genetic materials) |  |
|  |  |  | Cultivated plants (including fungi, algae) grown as a source of energy |  |
|  |  |  | Plants cultivated by in- situ aquaculture grown for nutritional purposes |  |
|  |  |  | Fibres and other materials from in-situ aquaculture for direct use or processing (excluding genetic materials) |  |
|  |  |  | Plants cultivated by in- situ aquaculture grown as an energy source |  |
|  |  | Reared animals for nutrition, materials or energy | Animals reared for nutritional purposes | Biomass component of material footprint and ecological footprint |
|  |  |  | Fibres and other materials from reared animals for direct use or processing (excluding genetic materials) |  |
|  |  |  | Animals reared to provide energy (including mechanical) |  |
|  |  | Reared aquatic animals for nutrition, materials or energy | Animals reared by in-situ aquaculture for nutritional purposes | Biomass component of material footprint and ecological footprint |
|  |  |  | Fibres and other materials from animals grown by in-situ aquaculture for direct use or processing (excluding genetic materials) |  |
|  |  |  | Animals reared by in-situ aquaculture as an energy source |  |
|  |  | Wild plants (terrestrial and aquatic) for nutrition, materials or energy | Wild plants (terrestrial and aquatic, including fungi, algae) used for nutrition | Not accounted for in any footprint |
|  |  |  | Fibres and other materials from wild plants for direct use or processing (excluding genetic materials) |  |
|  |  |  | Wild plants (terrestrial and aquatic, including fungi, algae) used as a source of energy |  |
|  |  | Wild animals (terrestrial and aquatic) for nutrition, materials or energy | Wild animals (terrestrial and aquatic) used for nutritional purposes | Not accounted for in any footprint |
|  |  |  | Fibres and other materials from wild animals for direct use or processing (excluding genetic materials) |  |
|  |  |  | Wild animals (terrestrial and aquatic) used as a source of energy |  |
|  | Genetic material from all biota (including seed, spore or gamete production) | Genetic material from plants, algae or fungi | Seeds, spores and other plant materials collected for maintaining or establishing a population | Not accounted for in any footprint |
|  |  |  | Higher and lower plants (whole organisms) used to breed new strains or varieties |  |
|  |  |  | Individual genes extracted from higher and lower plants for the design and construction of new biological entities |  |
|  |  | Genetic material from animals | Animal material collected for the purposes of maintaining or establishing a population | Not accounted for in any footprint |
|  |  |  | Wild animals (whole organisms) used to breed new strains or varieties |  |
|  |  | Genetic material from organisms | Individual genes extracted from organisms for the design and construction of new biological entities | Not accounted for in any footprint |
|  | Other types of provisioning service from biotic sources | Other | Other | Not accounted for in any footprint |
| Provisioning (Abiotic) | Water | Surface water used for nutrition, materials or energy | Surface water for drinking | Blue water footprint |
|  |  |  | Surface water used as a material (non-drinking purposes) |  |
|  |  |  | Freshwater surface water used as an energy source |  |
|  |  | Ground water for used for nutrition, materials or energy | Ground (and subsurface) water for drinking |  |
|  |  |  | Ground water (and subsurface) used as a material (non-drinking purposes) |  |
|  |  |  | Ground water (and subsurface) used as an energy source |  |
|  | Non-aqueous natural abiotic ecosystem outputs | Mineral substances used for nutrition, materials or energy | Mineral substances used for nutritional purposes | Material footprint |
|  |  |  | Mineral substances used for material purposes |  |
|  |  |  | Mineral substances used for as an energy source |  |
|  |  |  | Non-mineral substances or ecosystem properties used for nutritional purposes (code 4.3.2.1) | Not accounted for in any footprint |
|  |  |  | Non-mineral substances used for materials (code 4.3.2.2) |  |
|  |  |  | Wind energy | Energy and exergy footprint |
|  |  |  | Solar energy |  |
|  |  |  | Geothermal |  |
